# Investigating the highest melting temperature materials: A laser melting study of the TaC-HfC system

**DOI:** 10.1038/srep37962

**Published:** 2016-12-01

**Authors:** Omar Cedillos-Barraza, Dario Manara, K. Boboridis, Tyson Watkins, Salvatore Grasso, Daniel D. Jayaseelan, Rudy J. M. Konings, Michael J. Reece, William E. Lee

**Affiliations:** 1 Centre for Advanced Structural Ceramics, Department of Materials, Imperial College London, South Kensington, London, SW7 2AZ, UK; 2 European Commission, Joint Research Centre, Institute for Transuranium Elements, P.O. Box 2340, D-76125 Karlsruhe, Germany; 3 Utah State University, Department of Mechanical and Aerospace Engineering, Logan, Utah, USA; 4 School of Engineering and Materials Science, Queen Mary University of London, Mile End Road, London E1 4NS, UK

## Abstract

TaC, HfC and their solid solutions are promising candidate materials for thermal protection structures in hypersonic vehicles because of their very high melting temperatures (>4000 K) among other properties. The melting temperatures of slightly hypostoichiometric TaC, HfC and three solid solution compositions (Ta_1−x_Hf_x_C, with x = 0.8, 0.5 and 0.2) have long been identified as the highest known. In the current research, they were reassessed, for the first time in the last fifty years, using a laser heating technique. They were found to melt in the range of 4041–4232 K, with HfC having the highest and TaC the lowest. Spectral radiance of the hot samples was measured *in situ*, showing that the optical emissivity of these compounds plays a fundamental role in their heat balance. Independently, the results show that the melting point for HfC_0.98_, (4232 ± 84) K, is the highest recorded for any compound studied until now.

The design of next generation hypersonic flight vehicles has raised interest in the discovery and development of materials that can operate in extreme environments. Hypersonic vehicles are equipped with sharp nose tips and leading edges to maximize flight performance. However, very high temperatures and heating rates are produced at these surfaces due to extreme velocities (>5 Mach). Thermal protection structures are required that can operate in air at temperatures that can exceed 2200 K, thus components are required to have very high melting temperatures^1^,^2^. The extreme conditions required for hypersonic applications have introduced the motivation for research and development of high temperature materials, including a group of ceramics commonly known as ultra-high temperature ceramics (UHTCs). A common definition of a UHTC is that of a ceramic that has a melting temperature above 3300 K. From a wide selection of materials that an engineer can choose only a limited number have melting temperatures above this criterion^3^. Tantalum carbide (TaC) and hafnium carbide (HfC) are of particular interest due to their high melting temperatures (>4000 K) which are the highest reported among all known inorganic materials^1^,^2^,^3^. They are known to form a continuous solid solution over the whole range of compositions.

The measurement of thermophysical properties at such high temperatures is difficult due to increased reactivity of the materials, heat losses, volatility, and loss of mechanical strength, particularly in the carbides of interest in this work. Several experimental investigations of the high temperature behaviour of pure and mixed tantalum and hafnium carbides were carried out mostly between 1930 and 1969, including the early work by Agte and Althertum^4^ and the comprehensive experimental campaign provided by Rudy^5^. In all these cases, high temperature melting of these carbides was studied with the help of the so-called Pirani-Alterthum approach^4^. This experimental method consists in observing the disappearance, in a sample heated by Joule effect, of a blackbody hole when it is filled by a newly formed liquid. Such an approach has been shown to be effective in many cases. However, it does involve significant uncertainty, especially at temperatures above 2200 K, where observation of liquid formation is extremely hard, in particular when using the early pyrometers that had limited time resolution.

Agte and Altherthum^4^ reported a maximum melting temperature (*T*_m_) for Ta_0.8_Hf_0.2_C of 4213 K, and this was later confirmed by Andriievski *et al*.^6^ who measured it as 4263 K. The temperature trend with a maximum melting point at an intermediate composition was not confirmed in the work by Rudy^7^. Rudy reported that the highest melting temperature compound is TaC_0.88_ at 4256 K, with a decreasing trend in *T*_m_ as the HfC concentration increased and a *T*_m_ of 4201 K for HfC_0.94_. In addition, the work by Gusev^8^ reports a calculated phase diagram (CALPHAD) for the TaC-HfC system in which the melting temperatures of the single member carbides are higher than those of the solid solutions, with TaC as the highest melting compound at 4275 K and 55 K higher than HfC. More recently, Hong and van de Walle^9^ predicted the melting temperatures using density functional theory (DFT) and their calculations suggest that the melting temperature of HfC_0.81_ is 3962 K, higher than TaC_0.88_ at 3830 K. Also these calculations were consistent with a local maximum within the solid solutions, for a composition close to Ta_0.8_Hf_0.2_C at 3920 K. The work of Hong and de Walle was particularly useful, in that it identified precise physical mechanisms behind the effects of carbon hypostoichiometry and alloying on the melting temperature of a given composition. In addition, HfC_0.56_N_0.38_ was reported as the compound with the highest melting temperature at 4141 K. In summary, the uncertain and contradictory results on the melting points reported suggest that: (i) thanks to the entropic contribution of lattice defects to reducing the free energy of the solid phase, slightly hypostoichiometric monocarbides might have melting temperatures higher than their solid solutions^7^,^8^,^9^; (ii) because of the Fermi energy position in the mixed carbides, a maximum melting temperature within the solid solutions (TaC-HfC) might exist^4^,^6^,^9^. This calls for further experimental work perhaps with other techniques than were previously available.

The laser melting technique used in this work constitutes an alternative to the Pirani-Alterthum method for studying the melting temperatures of the Ta-Hf-C compounds. It allows precise control of the time for which the sample is kept at extreme temperatures, which can be reached in the order of milliseconds if need be. Experiments of subsecond duration address several of the challenges associated with measurements at very high temperatures. The laser pulse shape and duration can be optimised in order to produce the desired heating and cooling rates while avoiding or at least minimising undesired effects such as sample vaporisation and reaction with the container. A pressure cell filled with an inert buffer gas at a moderate pressure of about 0.3 MPa is used to slow down the evaporation from the sample surface and prevent coating of the windows. Millimetre long graphite screws hold the sample, so that interaction with the container is kept to a minimum. In addition, only the central region of the sample surface is typically melted. This is then surrounded by solid material in both the radial and axial directions. The surrounding solid part of the sample can be considered to form a kind of “self-crucible” insulating the area of interest from the sample holder and preventing contamination with foreign material. A fast pyrometer with a time resolution of the order of 10 μs and spectro-pyrometer with a time resolution of the order of 1 ms are used to record the sample thermal radiance in the visible and near infrared spectral range.

The laser melting technique has been successfully used to study several refractory systems such as uranium carbides^10^, uranium oxides^11^, plutonium oxides^12^,^13^, uranium nitrides^14^ and zirconium carbides^15^,^16^. The results obtained on compounds with already well-assessed phase transition temperatures are in good agreement with the literature data.

## Rationale and experimental approach

**Figure 1:**
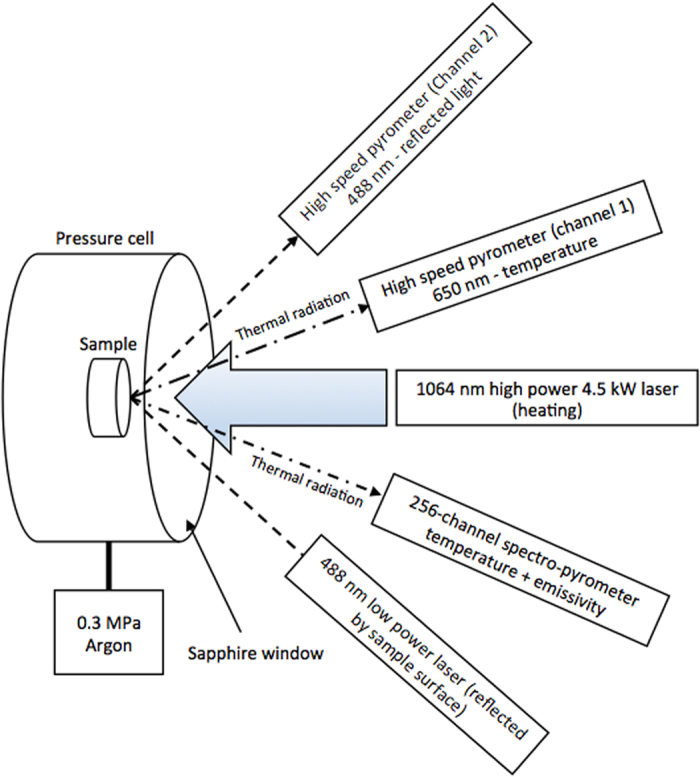
Schematic of the experimental laser-heating set-up.

The current experiments were carried out using a laser-heating technique, as described in detail by Manara *et al*.^11^,^17^. A 4.5 kW Nd:YAG cw laser (HLD4506, TRUMPF, Schramberg, Germany) was used for heating samples beyond melting on a circular area of 5 mm in diameter. A schematic of the experimental laser-heating set-up is shown in Fig. 1. Radiation pyrometers are the only devices available for the measurement of temperatures exceeding 2500 K. They were used in the present work and in the previous investigations on the same materials^4^,^6^,^7^. They measure at one or more wavelengths the sample radiance, which is the electromagnetic radiation power density per unit surface, wavelength and solid angle thermally emitted by the sample at a given temperature. It is linked to the sample surface temperature through Planck’s law of blackbody radiation (Equation 1):


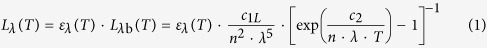


*L*_*λ*_ and *L*_*λ*b_ being the spectral radiance thermally emitted by the sample and a blackbody, respectively, at the ‘local’ wavelength *λ*, i.e. in a medium with refractive index *n*, and temperature *T*. *c*_1*L*_* = 2·h·c*_*0*_^2^ is the first radiation constant for radiance and *c*_2_ = *h·c*_0_*·k*_B_* = *14388 μm·K^18^ is the second radiation constant, with *c*_0_ denoting the speed of light in vacuum, *h* the Planck constant, and *k*_B_ the Boltzmann constant. A blackbody is a surface that absorbs all radiant flux of all wavelengths and polarisations incident upon it from all possible directions. For a prescribed temperature and wavelength, no surface can emit more thermal radiation than a blackbody, which in addition is an isotropically diffuse (lambertian) emitter. A blackbody is an ideal surface that can only be approximated in practice. The spectral-directional emissivity, *ε*_*λ*_ of a real surface is a measure of its ability to emit thermal radiation, as compared to that of a blackbody at the same temperature and wavelength. Therefore, it assumes values between zero and unity, the latter value corresponding to a blackbody. As the name implies, *ε*_*λ*_ will generally also depend on direction. For the particular case of emission in the direction normal to the surface it is called normal spectral emissivity (NSE). Pyrometers in the present work were set up in this direction and ‘emissivity’ will always refer to the NSE. The value of this parameter is related to the electronic properties and the surface morphology of the investigated material. The latter can however be neglected, in a first approximation, when one assumes as ideally flat the surface of a melting solid, or a freezing liquid like those studied here.

The value of the NSE determines how bright a surface appears compared to a blackbody at the same temperature and wavelength. The (spectral) radiance temperature of a surface is the temperature at which a blackbody emits the same amount of spectral radiance as the surface. As the NSE is smaller than one, non-blackbodies always appear less bright than a blackbody would at the same temperature and, therefore, the radiance temperature is always lower than the (true) temperature of the non-blackbody.

The sample temperature was measured with a fast two-channel pyrometer, using the first channel operating at about 655 nm and calibrated against a standard tungsten ribbon lamp at radiance temperatures up to 2500 K at the same wavelength. Calibration linearity was also verified using a graphite blackbody heated up to 3300 K. The second channel of this pyrometer was tuned to 488 nm, the wavelength of a lower power (1 W) Ar^+^ laser that was focussed on and reflected by the melting/freezing sample surface. This reflected light signal (RLS) was used to facilitate the detection of phase transitions by observing sudden changes and oscillations in the RLS, in parallel to the classical thermal arrest analysis directly carried out on the temperature vs. time curves. A 256-channel spectro-pyrometer operating between 488 nm and 1011 nm and radiometrically calibrated against a standard tungsten-ribbon lamp and a blackbody was employed for the study of the thermally emitted spectral radiance of the current samples. Because of the poor signal recorded by tail-detectors of this spectro-pyrometer, in practice only 185 channels between 515 nm and 900 nm were used in this work. This spectral range is well suited for the study of Planck’s curves around their maximum value for temperatures between 3000 K and 6000 K, according to Equation 1. Both pyrometers were focussed in the centre of the laser-heated sample surface, on sighting spots of 1 mm in diameter. Being the pyrometers’ sighting spots about twenty-five times smaller than the heated area, this geometry ensured that effects of thermal gradients at the edges of the laser spot were not affecting the pyrometer reading.

Since little data exists on the optical properties of tantalum and hafnium carbides, especially at extremely high temperatures, in the current work first approximation true temperature versus time curves (thermograms) were obtained starting from NSE values at 650 nm reported by Deadmore^19^ up to approximately 3075 K on pure and mixed Ta and Hf carbides synthesised by electric discharge machining of hot-pressed stocks. The latter NSE data were preferred to those measured by other authors^20^,^21^,^22^ because the samples investigated by Deadmore were produced with a more similar technique and had more similar carbon content to the current ones. For example, Danilyants *et al*.^21^ obtained lower NSE values on re-melted TaC_0.8_ samples. However, their data cannot be used as a reference for the current investigation, as they were obtained on samples with significantly less carbon, which obviously resulted in a more metallic behaviour of the NSE. It should be noted, as highlighted by Danilyants *et al*., that the sample preparation technique, carbon content and surface state can greatly affect the actual NSE of the carbides investigated in this work.

In order to shed some light on this complex matter, a test exercise was performed. Deadmore’s emissivity data at 3075 K were used as starting NSE values for fitting the current radiance spectra recorded with the spectro-pyrometer up to the melting temperature of each composition. In a first approximation, recommended by Neuer^23^, the fitting procedure was performed within the grey-body hypothesis, i.e. wavelength-independent NSE. Subsequently, more tests were performed assuming a decreasing NSE with increasing wavelength. Although this procedure is purely qualitative, NSE values within ±5% of Deadmore data at 3075 K yielded best fits of the current radiance spectra, up to the solid/liquid transition. Therefore, Deadmore’s NSE data were used in the present work with a conservative ±10% relative uncertainty bands.

## Results and Discussion

### Laser melting thermal analysis

**Figure 2:**
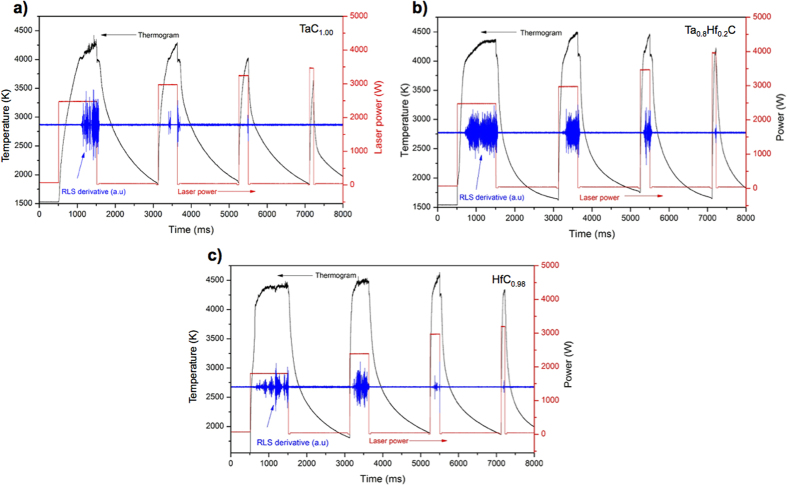
Thermogram, laser pulse profile and derivative of the RLS of laser melted. (**a**) TaC_1.00_; (**b**) Ta_0.8_Hf_0.2_C; and (**c**) HfC_0.98_. The thermogram (black line) shows the sample temperature, the blue signal is the RLS derivative in which phase transitions are detected by vibrations/oscillations in the signal. The red signal is the power output profile of the laser.

Laser heating experiment consisted of four consecutive laser pulses, starting with a low-power pulse held for a long hold time (typically 1000 ms), and then the power of the subsequent pulses were increased but shortened in hold time (typically few hundreds ms). The pre-heating stage and the successive pulses were conceived to minimise thermal stresses and the risk of mechanical failure of the samples. The output signals of a laser experiment for TaC_1.00_ are shown in Fig. 2a, the thermogram (black line) shows the sample temperature during the laser heating and cooling cycle, the power output profile of the laser is the red signal and the RLS signal (blue) shows that melting occurred during the first three laser pulses, while no phase transformations were observed for the last pulse. In order to produce a pronounced freezing arrest it was not enough to exceed the melting temperature momentarily. A sufficiently large mass had to be melted, for instance by using a longer laser pulse. Oscillations in the RLS and a thermal arrest in the cooling section of the thermogram confirmed that the sample had melted and re-solidified. Melting of the sample might be difficult to identify using only the thermogram, since, as predicted by numerical simulations of the experiments^11^, due to the highly non-uniform (surface) character of laser heating no thermal arrest is observed during fast heating. Only a weak change in the surface heating rate may be visible in these cases, caused by the progression of the melting front into the sample that consumes a part of the imparted laser energy as more material is melted. Therefore, the melting initiation is often easier to identify with the RLS signal. The noise observed after melting at the highest temperatures may in some cases be caused by boiling of the sample surface^10^. Since no further inflections were observed during heating or cooling it can be inferred that, within the current experimental uncertainties, TaC melts congruently and does not show further phase changes below the melting temperature. Further analysis of the clear solidification thermal arrest showed that the *T*_m_ of TaC_1.00_ was (4041 ± 77) K using the Deadmore NSE value of 0.52 with a relative uncertainty of 10%. Here and in the rest of this work, uncertainty bands are calculated using the error propagation law [11], considering as independent the uncertainties due to NSE, data dispersion and pyrometer calibration. During melting a significant volume of vapour was observed visually, which is related to the disturbed shape of the thermogram at the highest temperatures^14^. However, vaporisation was not a concern for the observed melting/freezing temperatures, as these showed satisfactory reproducibility with 12 laser shots performed on each sample with different peak pulse and duration. The experiment geometry was actually conceived in such a way that the vapour plume evolving parallel to the surface towards the upper and cooler parts of the disk would not interfere with the optical measurement of the temperature, performed on the hottest zone and perpendicularly to it. Sequences of four successive pulses were performed. The sample never cooled to a temperature lower than 1500 K between the pulses. This helped to reduce thermal stresses and preserve the mechanical integrity of the material throughout the tests. In the current tests, durations of the successive laser pulses were varied by a factor up to ten, and their powers by a factor up to two. Using laser pulses with different peak powers and durations was a good way to check that the solid/liquid transitions occurred at the thermodynamic equilibrium, and that the vapour formation had a negligible effect on the recorded melting/solidification temperatures. The observed phase transition temperatures were indeed independent, within the experimental uncertainty, of the laser pulse power and duration, whereas, of course, longer and more powerful laser pulses implied higher peak temperature, longer dwelling time beyond melting, larger vapour production etc. In order to carry out additional tests of possible vapour plume interference with the pyrometric temperature measurements, more laser shots were performed with higher peak power. In these tests (results not shown in this paper), maximum temperatures beyond 6000 K were attained for a few tens of ms, with a consequently large production of vapour. Nonetheless, even in these extreme cases, the solidification arrests were observed at the same temperatures as those recorded in more standard experiments, within uncertainty.

Similarly, Ta_0.8_Hf_0.2_C samples were tested under the same laser profiles as TaC_1.00_ (Fig. 2b). Comparing the profiles of TaC_1.00_ and Ta_0.8_Hf_0.2_C it was observed that at the same thermal conditions Ta_0.8_Hf_0.2_C melted more quickly than TaC_1.00_ (~300 ms sooner) at (4178 ± 82) K using the Deadmore NSE value of 0.47 with a relative uncertainty of 10%. At higher temperatures there was less disruption in the RLS signal compared to that of TaC, and subsequently less vaporisation during melting. After the end of the laser pulse the sample was cooled naturally and a slight undercooling effect was produced during the thermal arrest. This effect is common during fast freezing conditions and is related to the solidification kinetics (nucleation and growth)^14^. It should be noted here, that the observed thermal arrests correspond to the solidus temperature, which is the most relevant for technological applications as it marks the onset of melting and the thermal stability limit of these materials. Unfortunately it has been impossible, with the current set-up and uncertainty, to distinguish a liquidus transition in the present thermograms. Similar experiments were conducted on Ta_0.5_Hf_0.5_C and Ta_0.2_Hf_0.8_C samples and the melting temperatures recorded were (4077 ± 78) K and (4120 ± 80) K based on the Deadmore NSE values of 0.55 and 0.56, respectively, and the same assumed NSE relative uncertainty of 10%.

**Figure 3:**
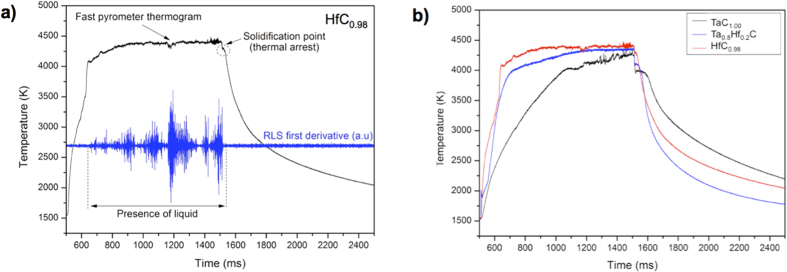
(**a**) Thermogram and RLS of laser melted HfC_0.98_ and (**b**) comparison of thermograms recorded during experiments performed on TaC_1.00_, HfC_0.98_ and Ta_0.8_Hf_0.2_C samples.

During the initial pulse of the HfC_0.98_ melting experiments it was observed that less laser power was required to melt hafnium carbide compared to samples of similar shape and volume of all the previous compositions (Fig. 2c). An initial laser pulse with an output power density of 256 MW·m^−2^ (1810 W on a circular focal spot of 3 mm diameter) was required to melt the sample, compared to 350 MW·m^−2^ required to melt TaC_1.00_. Melting of HfC_0.98_ samples was also achieved with intermediate and short pulses. The lower thermal diffusivity^24^ of HfC compared to TaC produces higher heat concentration in the sample surface, producing higher temperatures at lower applied power. Furthermore, much less vaporisation was observed in pure hafnium carbide compared with all of the other compositions, judging from the size of the vapour plume traces visible on the sample holder above the sample after the melting experiments. Most probably the excess power needed to heat the other compositions was largely dissipated in the sample vaporisation. However, higher temperatures were necessary to melt the HfC samples. A change in slope in the thermogram was observed as the sample started to melt (Fig. 3a), with less vaporisation observed during the melting stage and with a clear thermal arrest during the cooling stage. With the Deadmore NSE value of 0.5 and a relative uncertainty of 10%, the melting temperature measured for HfC_0.98_ was (4232 ± 84) K, the highest value measured in the Ta-Hf-C system. A comparison between the thermograms of the first laser shot on HfC, TaC, and Ta_0.8_Hf_0.2_C is shown in Fig. 3b.

**Figure 4:**
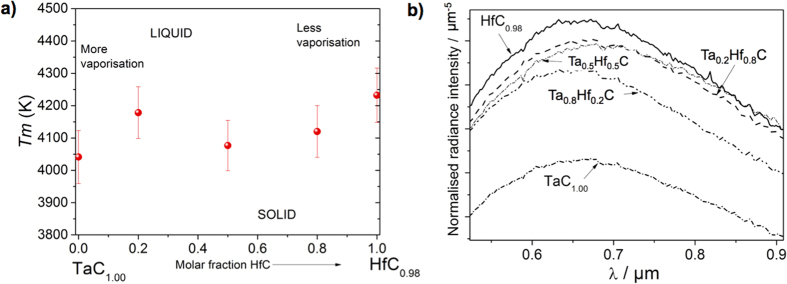
(**a**) Experimental melting temperatures (*T*_m_) obtained by correcting the current radiance temperatures with Deadmore’s emissivity data in the TaC-HfC system as a function of HfC content, and (**b**) normalised thermal radiance measured by a multi-channel spectro-pyrometer at the freezing temperatures of the five compositions studied in this work.

The results are summarised in Fig. 4a. They reveal that, within the uncertainty of the Deadmore NSE values, there could indeed exist a local maximum melting point of the solid solutions near the Ta_0.8_Hf_0.2_C composition, at 4178 K, in fair agreement with the value reported by Agte and Alterthum^4^. However, this maximum melting point is only local, because the maximum observed melting point in the Ta-Hf-C system is for HfC_0.98_, at 4232 K. In addition, unlike previous experimental reports, the *T*_m_ of TaC measured in this study was the lowest in the Ta-Hf-C system at 4041 K. Intermediate compositions between Ta_0.8_Hf_0.2_C and HfC_0.98_ have higher solid/liquid transition temperatures than pure TaC. The highest melting temperature previously reported for HfC_0.94_ is 4201 K and the highest for any compound is 4256 K for TaC_0.88_, both temperatures determined by Rudy^5^. Gusev *et al*.^8^ reported a similar trend to Rudy’s results^5^ but with slightly higher values (Table 1), however their values were obtained from thermodynamic calculations. The trend obtained in our work is similar to the one predicted by Hong and van de Walle^9^ with HfC_0.81_ having the highest melting temperature, TaC_0.88_ the lowest, and with a local maximum for Ta_0.8_Hf_0.2_C.

Figure 4b reports the radiance spectra recorded between 515 nm and 900 nm at the solidification arrest for the investigated compositions. They span most of the visible range where, according to Planck’s Equation 1, the peak value and a significant part of the total thermal radiance is emitted^25^ at the current melting/solidification temperatures and they include the wavelength of peak thermal radiance at these temperatures. Interestingly, the solidification-temperature radiance spectra follow only partially the trend reported in Fig. 4a for the melting/freezing temperatures calculated from the measured radiance temperatures at 650 nm and the NSE values reported by Deadmore. At the respective melting/solidification points, pure hafnium carbide does emit the highest radiance and pure tantalum carbide the lowest, which is consistent with the temperature data reported in Fig. 4a. However, in terms of solidification radiances below 700 nm, the mixed compositions display a rather monotonic increase going from Ta-richer compositions to Hf-richer ones. Therefore, the existence of a local maximum melting temperature for Ta_0.8_Hf_0.2_C is not obvious from the radiance spectra. In addition, the monotonic trend of spectral radiance vs. composition seems to be disrupted at longer wavelengths by the radiance spectrum of Ta_0.5_Hf_0.5_C at its solidification arrest, which first exceeds that of Ta_0.8_Hf_0.2_C and then that of HfC at wavelengths longer than about 700 nm and 850 nm, respectively. The reason must lie with the dependence of the NSE on composition and wavelength and no definite conclusions can be drawn on the absolute melting/solidification temperatures without a direct *in-situ* measurement of NSE, an extremely challenging task when one considers the extreme temperatures involved. On the other hand, the observed link between sample emissivity and the existence of a local maximum melting/solidification temperature can be taken as an additional, experimental hint in favour of the hypothesis held by Hong and van de Walle^9^, that the position of the Fermi level in Ta-Hf-C compositions has a direct effect on their melting point. This should certainly encourage further experimental research in line with Hong and van de Walle’s theoretical prediction, based also on considerations on the liquid phase entropy, that mixed Ta-Hf-C-N compositions might display even higher melting points than those assessed for the ternary Ta-Hf-C system.

Finally, the current results displayed in Fig. 4a and b ensure that pure hafnium carbide can be retained the compound emitting the highest radiative power intensity at its the melting temperature, at least in the spectral range investigated here. This is certainly most useful information in view of its employment as a refractory material.

### Post-melting characterisation

**Figure 5:**
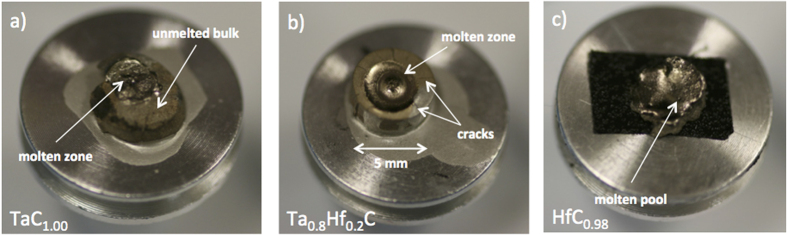
Photographs of laser-melted samples. (**a**) TaC_1.00_; (**b**) Ta_0.8_Hf_0.2_C; and (**c**) HfC_0.98_.

The samples were characterised after the melting experiments. A photograph of post-melted samples is shown in Fig. 5. After 8 laser pulses the TaC_1.00_ sample (Fig. 5a) showed separation of material from the bulk material around the molten pool, a bright melted and refrozen pool at the centre of the sample was observed. Furthermore the Ta_0.8_Hf_0.2_C pellet (Fig. 5b) showed better mechanical resistance to the laser pulses than the other compositions, the sample retained its shape even after 12 consecutive laser shot cycles. A rounded region of a molten pool of ca. 3 mm with a swollen region at the centre of the sample was observed. In addition, radial cracks initiating at the focal spot of the laser and propagating to the edge of the sample were produced. Massive damage was produced on the HfC_0.98_ sample during the laser melting experiments. A molten pool covering the whole surface with a crater-like shape is shown in Fig. 5c. During laser heating, parts of the sample separated and fell off the sample, due to high porosity (about 15%). In some experiments, the thermogram showed disruptions due to bulk material falling off the sample surface. This was stabilized after a few laser shots, when a denser surface was produced after melting and freezing of the material.

**Figure 6:**
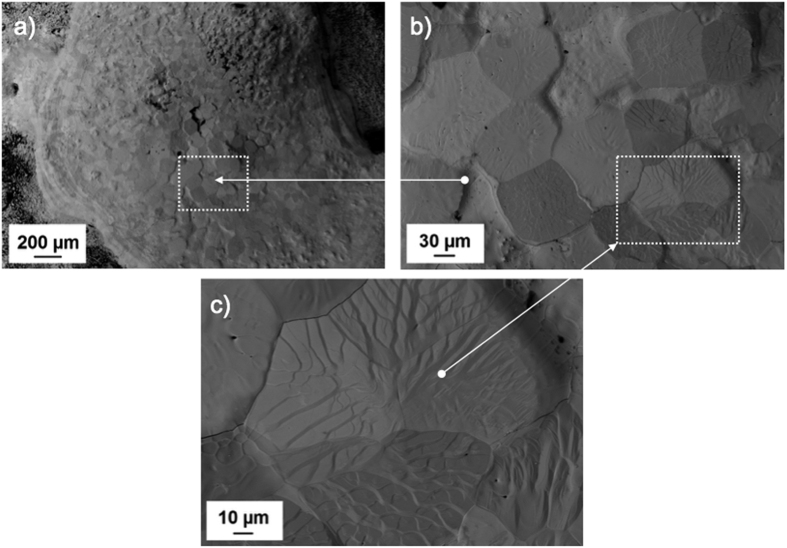
BSE image of TaC_1.00_ after laser induced melting showing. (**a**) molten pool; (**b**) morphology of grains after melting; and (**c**) higher magnification of a grain after melting.

In addition, SEM analysis was conducted on post-melted samples. A BSE image in Fig. 6a shows a melted sample of TaC_1.00_ after laser heating experiments. The centre of the sample consists of a molten pool surrounded by unmelted bulk material. At the centre of the molten pool a crater can be observed where the focal point of the laser was targeted. A region surrounding the void or crater (Fig. 6b) shows the recrystallised grains after freezing. The morphology of the grains shows a rippled surface after freezing. This morphology is uniform through the molten pool and a single grain can be observed at higher magnification in Fig. 6c.

**Figure 7:**
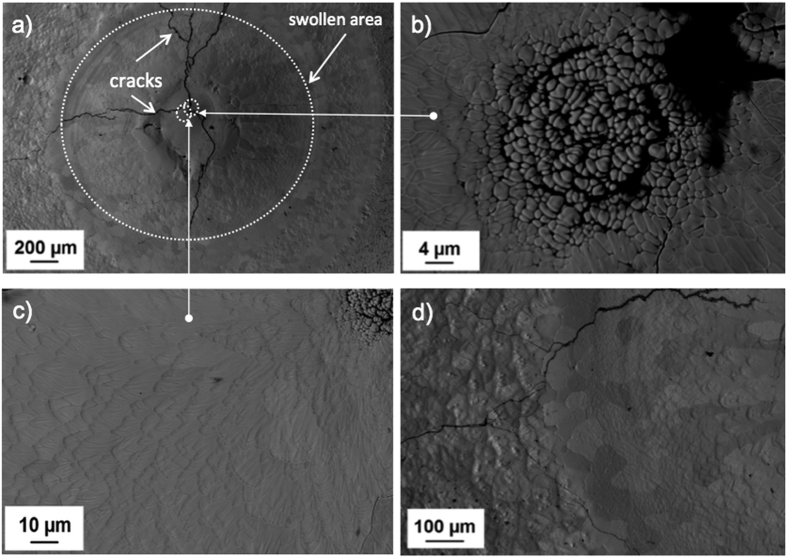
BSE image of the solidified molten pool of Ta_0.8_Hf_0.2_C; (**a**) swollen area near the centre of the laser focal point with cracks in the surface; (**b**) dendritic microstructure after repeated melting; (**c**) ripples at the surface near the laser focal point; and (**d**) change in grain morphology from the swollen area to a heat affected zone.

The molten region of a Ta_0.8_Hf_0.2_C sample is shown in Fig. 7a revealing a circular region of molten material with a swollen area at the centre. Cracks were observed starting from the focal point of the laser, radially propagating to the outer regions of the sample. A dendritic structure was formed after consecutive melting of the same spot. In Fig. 7b next to the dendritic structure cracks have formed. As the material surrounding the dendrites froze, ripples formed on the surface of recrystallised grains (Fig. 7c). Also revealed is a change in grain morphology from the swollen to a heat affected zone (i.e. not melted), with three different areas; elongated grains with rippled surface, then a transition zone with equiaxed grains, and finally a region with grains with lumps or peaks at each grain (Fig. 7d).

**Figure 8:**
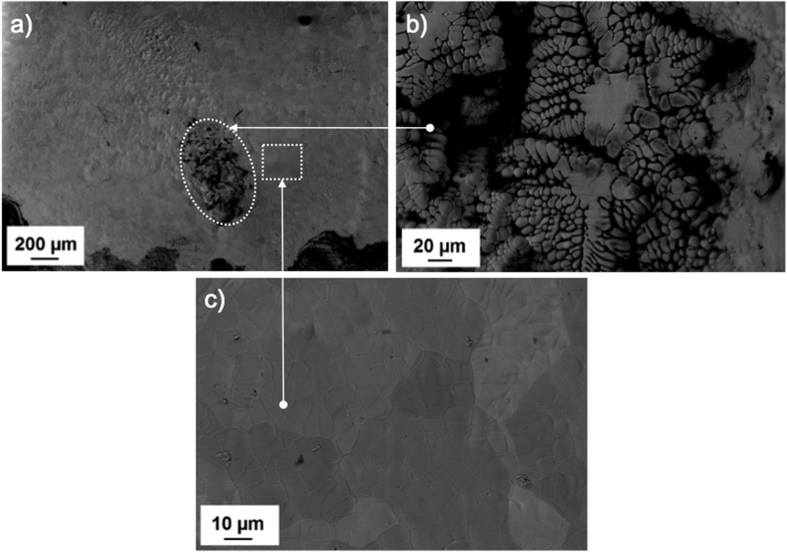
BSE image of molten pool of HfC_0.98_: (**a**) molten pool; (**b**) formed dendrites after repeated melting; and (**c**) grain morphology around dendritic structure.

BSE image of melted HfC_0.98_ shows in Fig. 8a the molten pool of the sample that constitutes the whole top surface of the sample after severe damage caused by repeated melting experiments. The area indicated by a white oval (Fig. 8a) shows the beam focal point where the highest thermal load was concentrated. It has a dendritic structure formed after consecutive melting and freezing of the same spot. An enlarged image (Fig. 8b) shows the formed dendrite structures with interdendritic voids shown in black contrast. The square indicated in Fig. 8a and enlarged in Fig. 8c shows the melted surface outside the dendritic region in which recrystallised equiaxed grains formed after freezing of the melted surface. This morphology is uniform throughout the molten pool surrounding the dendritic region.

**Figure 9:**
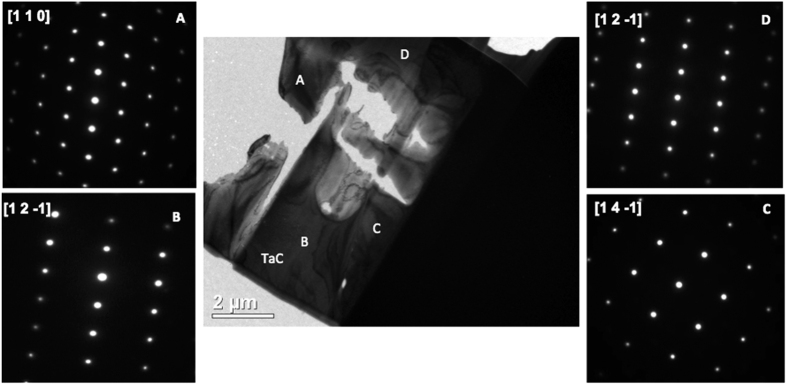
BF TEM image of FIB section of laser melted TaC_1.00_ and respective SAED indexed patterns showing cubic grains.

An important concern in melting experiments is the occurrence of phase transformations other than the solid-liquid one. Due to vaporisation of carbon products and a possible reduction in carbon content it is possible that hexagonal Ta_2_C or other Ta-C phases formed. This situation is not a concern in HfC where the carbide has a wide range of non-stoichiometry at high temperatures and does not form lower order carbides^5^. Additional TEM analysis (Fig. 9) conducted on FIB prepared sections showed that in a melted TaC_1.00_ sample only TaC grains were observed. SAED patterns were indexed, and only cubic [1 1 0], [1 2 −1] and [1 4 −1] zone axes were identified, confirming that the grains analysed were cubic TaC. No lower temperature thermal arrests were observed in the thermograms of the present study. Such thermal arrests would have been expected, according to the phase diagram proposed by Rudy^5^ if new phases, such as Ta_2_C, had been produced as a result of a hypothetical massive vaporisation of carbon-rich species.

## Concluding remarks

The results from this work show that HfC_0.98_ has the highest melting point in the TaC-HfC system at (4232 ± 84) K, which make it the highest melting point compound, since the melting temperature of TaC was experimentally found to be lower than HfC in this work.

In mixed compositions, a local maximum melting temperature was observed for the Ta_0.8_Hf_0.2_C, in agreement with previous research. Nevertheless, the current melting temperature of HfC_0.98_ remains the highest of the series. The presence of a local maximum melting temperature for mixed Hf-Ta carbides has been shown to be accompanied by a spectral emissivity effect, consistent with the existence, recently proposed by Hong and van de Walle^9^, of a link between the melting behaviour and the Fermi level position in this kind of compounds. This should boost further experimental research on Ta-Hf-C-N compositions that might display even higher melting temperatures than those assessed for the ternary Ta-Hf-C system, in line with Hong and van de Walle’s theoretical predictions.

## Methods

Commercial powders of TaC (99.5 wt%, −325 mesh; ABCR, Karlsruhe, Germany) and HfC (99 wt%, −325 mesh; ABCR, Karlsruhe, Germany) were used as starting materials. Laser diffraction was used to measure the particle size distribution of the starting powders (Mastersizer 2000, Malvern; Worcestershire, UK). The measured particle size of the as-received powders was *d*_50_: 3.44 ± 0.07 μm for TaC and *d*_50_: 2.0 ± 0.10 μm for HfC. The carbon, sulphur, nitrogen and oxygen contents of the sintered products were determined by combustion analysis using a carbon-sulfur elemental mass induction analyser (EMIA) (Horiba, Longjumeau, France) and an elemental mass gas analyser (EMGA) (Horiba, Longjumeau, France). The elemental analysis of TaC and HfC powders after sintering are presented in Table 2. From this analysis the compositions measured for the sintered products were TaC_1.00_ and HfC_0.98_. Solid solution compositions were prepared by mixing appropriate amounts of powders using a rotary mill (CAPCO Test Equipment 12VS, Ipswich, UK) with SiC media for 24 h in ethanol. The powders were dried in a rotary evaporator (Rotavapor R124, Buchi; Flawil, Germany) under vacuum. In addition, powders were lightly ground in a mortar with a pestle and sieved to a final mesh size of 50 μm. Densification of the powders was conducted using an SPS furnace (FTC HP D25; FCT Systeme GmbH, Rauenstein, Germany). 15 g of the powder mixtures was poured into a 20 mm diameter graphite die which was lined with 0.3 mm-thick graphite foil to maximise electrical and thermal conduction between the punches and the die. To reduce heat loss, a graphite felt was placed around the die. The sintering schedule used for pure HfC and TaC was 2373 K for 20 min at 60 MPa. For the solid solution compositions (Ta_0.8_Hf_0.2_C, Ta_0.5_Hf_0.5_C and Ta_0.2_Hf_0.8_C) a two-step sintering schedule was used to achieve homogeneous solid solutions. The sintering conditions were 2373 K for 20 min and 55 MPa, and a second step at 2623 K for 20 min and 32 MPa. After sintering the density of the samples was measured using Archimedes’ method with distilled water as the immersion fluid. The relative density of the samples was 98.3% for TaC_1.00_, 97.7% for Ta_0.8_Hf_0.2_C, 95.7% for Ta_0.5_Hf_0.5_C, 87.0% for Ta_0.2_Hf_0.8_C and 85.3% for HfC_0.98_. Subsequently, samples were polished in successive steps using diamond discs and slurries to a 1 micron surface finish.

For the laser melting experiments, samples of 5 mm diameter and 5 mm thickness were cut by electrical discharge machining (EDM) from the 20 mm diameter sintered pellets. Samples were held in a graphite fixture inside the pressure cell fitted with a sapphire window. For each experiment the cell was evacuated using a vacuum pump and backfilled with argon at a 0.3 MPa pressure. The laser beam was focused on the specimen surface through an optical fibre and with the help of a low-power He-Ne pilot laser. The target position was adjusted so that the beam hit the centre of the sample. A summary of the laser pulse profiles and power density is shown in Table 3.

Microstructural analysis of post-melted samples was performed by scanning electron microscopy (SEM) (Auriga, Carl Zeiss; Oberkochen, Germany) and images were taken at an acceleration voltage of 15 kV. Transmission electron microscopy (TEM) samples were cut out from specimens using a focused ion beam (FIB) instrument (Helios 650 Nanolab, FEI, Hillsboro, OR, USA) and TEM images and SAED patterns were taken using a JEOL FX2100 (Tokyo, Japan) at 200 kV. Selected Area Electron Diffraction (SAED) patterns of zone axes were recorded. The SAED patterns were indexed using SingleCrystal analysis software (CrystalMaker Software Ltd.; Begbroke, UK).

### Data Availability

All data generated during the course of this research is contained within this thesis and can be accessed via http://hdl.handle.net/10044/1/32138.

## Additional Information

**How to cite this article**: Cedillos-Barraza, O. *et al*. Investigating the highest melting temperature materials: A laser melting study of the TaC-HfC system. *Sci. Rep.* **6**, 37962; doi: 10.1038/srep37962 (2016).

**Publisher's note:** Springer Nature remains neutral with regard to jurisdictional claims in published maps and institutional affiliations.

## Figures and Tables

**Table 1:** Reported melting temperatures (*T*_m_) in the TaC-HfC system including the results of this work.

Authors	Melting temperature *T*_*m*_ (K)							
	Ref.	Year	TaC (C/Ta)	Ta_0.8_Hf_0.2_C	Ta_0.5_Hf_0.5_C	Ta_0.2_Hf_0.8_C	HfC (C/Hf)	Method
Agte and Alterthum	4	1930	4150	4213	—	—	4160	Pirani-type furnace
Rudy	7	1965	4256 ± 15 (0.88)	4238	4218	4207	4201 ± 20 (0.94)	Pirani-type furnace
Andrievskii *et al*.	6	1967	4113 (0.98)	4263	—	—	4023 (0.97)	Pirani-type furnace
Gusev *et al*.	8	1985	4275^*^	4233^*^	4190^*^	4210^*^	4221^*^	CALPHAD
Okamoto	26	1998	4242^*^ (0.88)	—	—	—	—	CALPHAD
Okamoto	27	2001	—	—	—	—	4215^*^ (0.94)	CALPHAD
Hong and van de Walle	9	2015	3830^†^ ± 24 (0.81)	3895^†^ ± 16	3859^†^ ± 14	3920^†^ ± 16	3962^†^ ± 27 (0.81)	DFT
This work	—	2016	4041 ± 77 (1.00)	4178 ± 82	4077 ± 78	4120 ± 80	4232 ± 84 (0.98)	Laser heating under containerless conditions and controlled atmosphere

(^*^By thermodynamic calculation, ^†^by DFT simulation).

**Table 2:** Composition of sintered powders of TaC and HfC from combustion experiments.

Compound	Wt%C	Wt%O	Wt% N	C/M Ratio (M = Ta, Hf)
TaC (sintered)	6.31 ± 0.03	0.37 ± 0.01	0.016 ± 0.001	1.00
HfC (sintered)	6.11 ± 0.05	0.49 ± 0.01	0.58 ± 0.005	0.98

**Table 3:** Laser pulse profiles for melting experiments of TaC-HfC ceramics.

Timescale	Hold time (ms)	Peak power (W)	Power density (MW/m^2^)
Long	1000	1810–2480	256–350
Intermediate-long	500	2400–2980	339–421
Intermediate-short	250	2980–3480	421–492
Short	100	3200–3980	452–563
